# Culturally appropriate patient-provider communication methods for Black women with breast cancer: a scoping review

**DOI:** 10.1007/s00520-025-09425-6

**Published:** 2025-04-21

**Authors:** Diana C. Litsas, Kirsten Paulus, Travis Nace, Ariel Hoadley, Yaara Zisman-Ilani, Laura A. Siminoff

**Affiliations:** 1 Department of Social and Behavioral Sciences, College of Public Health, Temple University, 1301 Cecil B. Moore Ave, Philadelphia, PA 19122 USA; 2 Temple University Health Sciences Library, 3500 N. Broad Street, Philadelphia, PA 19140 USA; 3 Johns Hopkins School of Medicine, 733 N Broadway, Baltimore, MD 21205 USA

**Keywords:** Health communication, Health decision-making, Breast cancer, Minority health

## Abstract

**Purpose:**

Black women with breast cancer experience a 40% higher mortality rate compared to white women, and this disparity may be influenced by suboptimal patient-provider communication. Evidence has suggested that Black patients with breast cancer have unique informational needs, yet few studies focus on patient-provider communication for this community. The aim of this scoping review was to identify best practices for communicating breast cancer diagnosis and treatment options with Black women.

**Methods:**

Following PRISMA-ScR, a search strategy was developed and implemented in 4 databases and grey literature. Coders achieved reliability and independently screened articles by title and abstract, then full-text. Articles were included if they studied Black patients with breast cancer and reported on patient-provider communication. Outcomes of interest included patients’ appraisals of their communication, and associated health outcomes.

**Results:**

A final sample of 27 studies were included. Black patients’ positive appraisal of their providers was based on their interactions, rather than providers' perceived expertise. Patients had negative appraisal of communication when they received limited information regarding their treatment plan, side-effects, or possibility of disease recurrence. High-quality communication was associated with adherence to adjuvant therapy; low-quality communication was associated with treatment discontinuation or delay, and lower self-rating of physical well-being.

**Conclusion:**

Patients’ perceptions of their quality of communication with their healthcare providers was associated with their treatment decisions and health outcomes. Future research is needed to test interventions that optimize communication between Black breast cancer patients and their providers, including discussing navigating barriers to care.

## Introduction

Breast cancer is the second leading cause of cancer death among women nationwide [1], with over 42,000 deaths in 2022 [2]. American women have a one in eight chance of developing breast cancer in their lifetime [3]. Furthermore, the incidence rates of female breast cancer increased by 1% every year between 2013–2022 [1]. A breast cancer diagnosis can begin when a tumor is detected on a mammogram, or when patients present with symptoms, such as a lump on the breast or change in breast appearance [3]. Advances in genetics and pathology have caused breast cancer staging to rapidly evolve, leading to more individualized and complex diagnoses [4]. Although gains have been made in treatment efficacy, with 90% of breast cancer patients surviving five years from diagnosis, a substantial disease burden persists [5]. A metanalysis found the risk of recurrence within five years after treatment was 17.2% [6], and this rate varies widely depending on the classification of disease and treatment pursued [7]. Even if breast cancer does not recur, undergoing breast cancer treatment can cause significant side effects in the short and long term, as well as social and psychological challenges.

Breast cancer incidence rates vary among different populations. While 90% of incidence cases are among women over 45 years of age [1], incidence rates have been rising among women under 45 years old [8]. This trend is especially concerning given that younger women have a greater risk of developing aggressive subtypes of breast cancer compared to older women [8]. Compared to other racial and ethnic groups, the highest incidence rates are among non-Hispanic white (139 cases per 100,000) followed by non-Hispanic Black (128.3 cases per 100,000) women [1].

Despite white women experiencing the highest incidence of the disease, Black women have a nearly 40% higher mortality rate (26.8 per 100,000) compared to white women (19.4 per 100,000). This is especially notable given evidence that these communities undergo surveillance screening at similar rates [9]. Efforts have been made to discern the cause of this disparity through examining the medical, biological, and genetic factors that can influence the prognosis of breast cancer. A potential influence that has received less attention is the finding that Black women with breast cancer report receiving inadequate information about their mammography results [9] and adjuvant endocrine therapy [10]. Given the sharp racial disparity in breast cancer outcomes, communication surrounding the diagnosis is worth further examination [11].

Communication is central to healthcare interactions and can equip patients with sufficient information about their conditions, enable them to make informed medical decisions, and promote treatment adherence [12]. Patients’ comprehension of their treatment plans is a facilitator for promptly pursuing care, while mistrust of the healthcare system and providers, as well as insufficient information about their treatment, are significant barriers [13]. Especially in the context of health disparities, equitable distribution of relevant information can be considered a critical need [14]. Medical practice norms that emphasize the value of interpersonal sensitivity and cultural competence can promote health communication that reduces racial disparities in the interpersonal aspects of healthcare [15]. Shared Decision-Making is a framework that acknowledges diversity in patient preferences, values, and barriers pertaining to healthcare [16]. It is a model of communication in which patients and their healthcare providers collaborate and exchange information so that patients are empowered to make an informed healthcare decision that reflects their beliefs and values [17]. Patient-provider communication that features components of Shared Decision-Making is associated with greater patient satisfaction and trust among patients with cancer [17]. Studies have found that patients’ trust in their physician is largely predicted by their physicians’ communication style—whether they actively listen, provide emotional support, offer clear information, and make time to answer questions [18]. Other evidence has shown that mistrust or suspicion of medical organizations is inversely related to communication ratings [19].

Optimizing the communication surrounding breast cancer diagnosis and treatment holds promise in mitigating patients’ informational barriers to promptly initiating treatment, and potentially improving clinical outcomes [20]. Evidence has demonstrated that higher adherence to breast cancer treatment was significantly associated with higher ratings of patient-provider communication [18, 19, 21]. Conversely, women have reported being deterred from initiating breast cancer treatment after experiencing unprofessional interactions [22]. Low quality communication can also increase the odds of unnecessary treatment, and decrease patient satisfaction [23].

While there is literature on health communication improving outcomes for other populations, few studies have explored the state of health communication among Black women with breast cancer. Even if studies include Black participants, they frequently report results in aggregate, without distinguishing the potentially unique needs of Black women. Furthermore, there is evidence of a differential effect of knowledge communicated by healthcare providers: white women were more likely to have increased knowledge from their physician following breast cancer diagnosis compared to Black women [24]. This finding is especially important given evidence that an increase in knowledge after breast cancer diagnosis is associated with patients’ active participation in treatment decisions [24]. The current state of literature has a limited understanding of communication between providers and Black patients with breast cancer, and how these interactions may manifest into severe racial disparities. There is a need to address this gap and determine what is known for communication with Black patients with breast cancer.

The research question of this scoping review is: Which patient-provider communication methods are culturally appropriate for Black women with breast cancer? The aim of this scoping review is to identify best practices for health communication regarding diagnosis and treatment plans, and to identify which elements can be improved to ensure that all patients have a thorough understanding of their condition as well as next steps for treatment.

## Methods

### Design

This review was conducted in accordance with the PRISMA-ScR guidelines [25]. A protocol was preregistered on the Center for Open Science (https://doi.org/10.17605/OSF.IO/8WE5G).

### Data collection

The review team worked with a biomedical librarian to develop detailed search strategies for each database using the PRISMA-ScR extension for search reporting. Based on a list of search terms provided by the team, the librarian (TN) developed the search for PubMed (NLM) and translated the search for every database searched. The PubMed (NLM) search strategy was reviewed by the research team to check for accuracy and term relevancy, and all final searches were peer-reviewed by another medical librarian following the PRESS Peer Review of Electronic Search Strategies checklist. The African American search hedge used in this search was borrowed from the African American Racial Disparities search hedge (1). The databases included in this search are PubMed (NLM), Embase (Elsevier), Web of Science Core Collection (Clarivate Analytics) and Applied Social Sciences Index and Abstracts (ProQuest) using a combination of keywords and subject headings. A grey literature search included Cochrane CENTRAL database (Wiley), TRIP Pro medical database (tripdatabase.com) and MedRxiv website (https://www.medrxiv.org). There were no limits to the search. All final searches were performed on June 13, 2022 by the librarian and were fully reported (TN). The full search strategies as reported by the librarian are provided in Appendix A.

### Inclusion criteria and article assessment

Studies were first screened by title and abstract, then full text by a single reviewer using Rayyan software (Cambridge, MA). Articles were included if they had a qualitative, quantitative, or mixed-methods study design; reviews and opinion pieces were excluded. Articles were included if they studied Black patients pursuing breast cancer treatment, and their communication with their healthcare providers. Communication for this study was operationalized to include live communication (e.g. in-person, over the phone, or via online call), as well as recorded communication (e.g., post mail, e-mail, voicemail, or text message). Studies were excluded if the results were reported in the aggregate, and it could not be discerned whether the results applied to Black women with breast cancer. Studies about patient experiences prior to (prevention measures) or following (survivorship) a breast cancer diagnosis were excluded.

Four reviewers achieved reliability by screening the same 10% of the total sample of study titles and abstracts (DCL, KP, AH). Once screening reliability was achieved, the coders independently screened the remaining articles by title and abstract; group consultation was sought for ambiguous articles. After reliability was achieved of screening the full-text of the article, two reviewers independently screened the remaining articles by full-text (DCL, KP).

Data were extracted from the final sample of selected articles. The primary outcomes were the topics which were reportedly discussed between Black patients and their healthcare providers, as well as patient appraisal of their communication with their healthcare providers. Secondary outcomes include patient health outcomes associated with reported patient-provider communication, including treatment decision-making, physical well-being, and obtained healthcare information. Other extracted data include study design, whether structured scales were implemented, sample size, patient stage of diagnosis, whether the provider role was specified (e.g. physician, nurse), whether patient-provider communication was observed synchronously or asynchronously, and author recommendations for future research.

## Results

The search resulted in 9,568 studies of which 4,877 duplicate studies were found and omitted by the librarian (TN) using the EndNote 20 duplicate identification strategy. This resulted in 4,519 records to screen from databases or registers and 172 records to screen from other methods, resulting in a total of 4,691 records. Duplicates were manually detected on Rayyan, resulting in 4,594 unique articles. Full search results are in Appendix B.

**Fig. 1:**
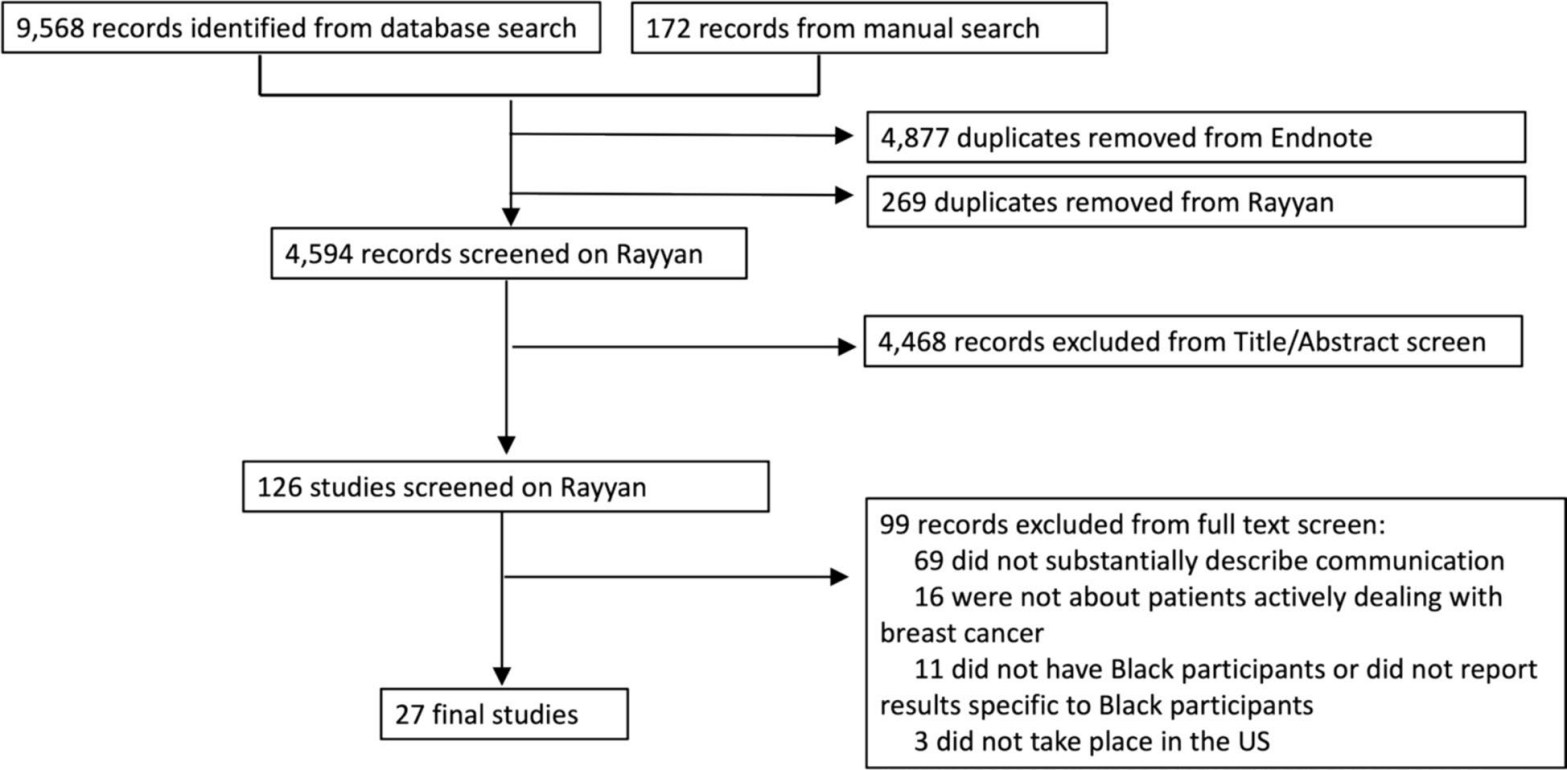
PRISMA flow diagram

The 4,594 articles were screened by title and abstract, yielding 126 studies. These were screened by their full text, and 99 articles were excluded: 69 did not explicitly describe the communication that took place between patients and their providers; 16 were not about patients actively managing their breast cancer; 11 did not report results specific to Black patients with breast cancer; 3 did not take place in the US (Fig. 1).

### Characteristics of selected articles

In total, the authors identified 27 studies that described communication between Black patients with breast cancer and their healthcare providers (Table 1). The total sample size of the included studies ranged from 9 to 4,002, and the sample size of Black participants ranged from 5 to 316. The proportion of Black participants in studies ranged from 6 to 100%. In one study, the exact sample size of Black participants was not reported.

**Table 1:** Characteristics of Included Studies (*n*=27)

First Author	Article Title	Publication Year	Study Type	Sample Size of Black Participants	Total Sample Size	Proportion of Black participants	Stages of Diagnosis Included in Sample
Campesino [26]	Surgical treatment differences among Latina and African American breast cancer survivors	2012	Phenomenological (Qualitative)	9	39	23%	I–IV
Chatman [22]	Addressing the unique psychosocial barriers to breast cancer treatment experienced by African-American women through integrative navigation	2011	Phenomenological (Qualitative); Post Program Assessment	69	69	100%	Not Specified
Check [27]	Understanding racial/ethnic differences in breast cancer-related physical well-being: the role of patient-provider interactions	2018	Prospective Cohort	316	4002	8%	I–IV
Davey [28]	"They paid no mind to my state of mind": African American breast cancer patients' experiences of cancer care delivery	2010	Phenomenological (Qualitative)	9	9	100%	I–III
Greene [29]	"This is some mess right here": Exploring interactions between Black sexual minority women and health care providers for breast cancer screening and care	2020	Phenomenological (Qualitative)	15	15	100%	Not Specified
Harper [30]	Incorporating Patient Satisfaction Metrics in Assessing Multidisciplinary Breast Cancer Care Quality	2015	*Non-Randomized Study	22	52	42%	I–IV
Hawley [31]	Latina patient perspectives about informed treatment decision making for breast cancer	2008	*Non-Randomized Study	227	877	26%	I–III
Heiney [32]	African American Women's Recollected Experiences of Adherence to Breast Cancer Treatment	2017	Narrative analysis	16	16	100%	Not Specified
Housten [33]	Movement Through Chemotherapy Delay to Initiation Among Breast Cancer Patients: A Qualitative Analysis	2022	Phenomenological (Qualitative)	5	22	23%	Not Specified
Kollie [34]	Evaluating racial differences in patient-provider decision making regarding treatment-related symptom management to advance supportive cancer care	2017	Phenomenological (Qualitative)	22	22	100%	Not Specified
Kwan [35]	Patient-physician interaction and quality of life in recently diagnosed breast cancer patients	2013	Prospective Cohort	110	1855	6%	Not Specified
Livaudais [36]	Breast cancer treatment decision-making: Are we asking too much of patients?	2012	Prospective Cohort	72	368	20%	I, II
Manfredi [37]	Are Racial Differences in Patient-Physician Cancer Communication and Information Explained by Background, Predisposing, and Enabling Factors?	2010	Cross-Sectional	132	245	54%	Not Specified
Samuel [38]	Equity in patient-provider communication regarding treatment-related symptoms and health-related quality of life (HRQOL) among breast cancer survivors.	2016	Focus Groups	Not specified	22	Not specified	Not Specified
Shelton [39]	Interpersonal influences and attitudes about adjuvant therapy treatment decisions among non-metastatic breast cancer patients: an examination of differences by age and race/ethnicity in the BQUAL study	2013	Prospective Cohort	181	1145	16%	I–III
Sheppard [40]	Disparities in Breast Cancer Surgery Delay: The Lingering Effect of Race	2015	Cross-Sectional	163	290	56%	I-III
Sheppard [23]	The role of patient-provider communication for black women making decisions about breast cancer treatment	2011	Phenomenological (Qualitative)	49	49	100%	I-III
Sheppard [41]	Development of decision-support intervention for Black women with breast cancer	2010	Phenomenological (Qualitative); Intervention Development	32	32	100%	I–IV
Sheppard [42]	Narrowing racial gaps in breast cancer chemotherapy initiation: the role of the patient-provider relationship	2013	Cross-Sectional	210	359	58%	Not Specified
Sheppard [43]	Reducing Racial Disparities in Breast Cancer Survivors' Ratings of Quality Cancer Care: The Enduring Impact of Trust	2016	Cross-Sectional	217	369	59%	Not Specified
Song [44]	Patient-Healthcare Provider Communication: Perspectives of African American Cancer Patients	2012	Grounded theory (Qualitative)	15	28	54%	I–IV
Sutton [19]	Medical Mistrust in Black Breast Cancer Patients: Acknowledging the Roles of the Trustor and the Trustee	2019	Cross-Sectional	210	359	58%	I–III
Torres [45]	Understanding the Breast Cancer Experience of Survivors: a Qualitative Study of African American Women in Rural Eastern North Carolina	2016	Cross-Sectional	32	32	100%	Not Specified
White-Means [46]	African American Women: Surviving Breast Cancer Mortality against the Highest Odds	2016	Phenomenological (Qualitative)	10	10	100%	Not Specified
White-Means [47]	Racial and Ethnic Disparities in Patient-Provider Communication With Breast Cancer Patients: Evidence From 2011 MEPS and Experiences With Cancer Supplement	2017	Cross-Sectional	72	239	30%	Not Specified
Wise [48]	Effects of using online narrative and didactic information on healthcare participation for breast cancer patients	2008	*Pre-Post Comparison	111	353	31%	I–IV
Yoon [49]	Symptom management after breast cancer treatment: is it influenced by patient characteristics?	2008	Cross-Sectional	50	448	11%	Not Specified

*Note:* All studies observational unless denoted “*”

About half of the included studies did not report patients’ Stage of diagnosis (*n* = 14). About a quarter of the studies (*n* = 6) included participants with diagnosis from Stage I through and inclusive of Stage IV, and about a quarter (*n* = 6) included participants with Stage I through III. The majority of articles studied communication between patients and their physicians (n = 21), while few articles studied communication between patients and their nurses, as well as their physicians (*n* = 5). For one study, the healthcare providers’ role was not specified. With the exception of one article [48], all included studies measured patient-provider communication asynchronously, rather than direct observation.

The vast majority of studies were observational (*n* = 24); few were intervention studies (*n* = 3). Of the observational studies, the most common study-types were interviews based in phenomenological methods (*n* = 8) and cross-sectional surveys (*n* = 8). Of the 15 included articles that administered surveys, several implemented the Group-Based Medical Mistrust Scale (*n* = 5), and the Makoul Communication Scale (*n* = 4), while a few implemented the Primary Care Assessment Survey (*n* = 3) and Patient Satisfaction Questionnaire (*n* = 2) (Table 2).

**Table 2:** Outcome measures of included studies (*n* = 27)

First Author	Publication Year	Type of Primary Outcome Measure (interview vs observation vs scale)	Name of Primary Outcome Measure	Type of Secondary Outcome Measure (interview vs observation vs scale)	Name of Secondary Measure
Campesino	2012	Instrument	NA_measured mastectomy vs lumpectomy	Interview	NA_perception of care delivery
Chatman	2011	Focus Group	NA_measured how managed health needs	Survey	NA_rating effectiveness of patient navigation program
Check	2018	Instrument	Functional Assessment of Cancer Therapy for Breast Cancer	Instrument	Interpersonal Processes of Care Survey
Davey	2010	Focus Group	NA_measured patient experiences of care	NA	NA
Greene	2020	Interview	NA_self-determination theory	NA	NA
Harper	2015	Instrument	NA_satisfaction with care quality	NA	NA
Hawley	2008	Instrument	Control Preferences Scale	NA	NA
Heiney	2017	Interview	NA_experience of treatment	NA	NA
Housten	2022	Interview	NA_barriers to chemotherapy	NA	NA
Kollie	2017	N/A	NA_shared decision making	NA	NA
Kwan	2013	Instrument	Inter- personal processes of care (IPC)	Instrument	Functional assessment of cancer therapy-breast cancer
Livaudais	2012	Instrument	NA_baseline treatment knowledge	Instrument	NA_decision regret
Manfredi	2010	Instrument	NA_interpersonal communication	Instrument	NA_Instrumental Communication
Samuel	2016	Interview	NA_patient-provider communication	NA	NA
Shelton	2013	Instrument	Mental Adjustment to Cancer	Instrument	Group- based Medical Mistrust Scale
Sheppard	2015	Instrument	Makoul Communication Scale	Instrument	Primary Care Assessment Survey; Group-Based Medical Mistrust
Sheppard	2011	Interview	NA_experience with breast cancer	NA	NA
Sheppard	2010	Interview	NA_factors affecting treatment decisions	NA	NA
Sheppard	2013	Instrument	Makoul Communication Scale	Instrument	Primary care assessment survey; race-based experiences scale; group-based medical mistrust scale
Sheppard	2016	Instrument	Patient Satisfaction Questionnaire Short Form	Instrument	Primary Care Assessment Survey; Group-Based Medical Mistrust Scale; Makoul Communication Scale; Communication Attitudinal Self-Efficacy Scale
Song	2012	Interview	NA_provider communication	NA	NA
Sutton	2019	Instrument	Group-Based Medical Mistrust Scale	Instrument	Makoul; Patient Satisfaction Questionnaire Short Form
Torres	2016	Interview	NA_experience of treatment	NA	NA
White-Means	2016	Interview	NA_experience of treatment	NA	NA
White-Means	2017	Instrument	Consumer Assessment of Healthcare Providers and Systems of the Medical Expenditure Panel Survey	NA	NA
Wise	2008	Instrument	Comprehensive Health Enhancement Support System	NA	NA
Yoon	2008	Instrument	NA_presence of symptoms	Instrument	SF- 12 physical component score

### Reported characteristics of patient-provider communication

Nearly all included articles reported patient-provider discussion of breast cancer management (*n* = 24), such as making treatment recommendations or explaining which treatment options were available [32]. Explanation of results/disease (*n* = 15) was also frequently reported, including physicians sharing information about patients’ test results as well as medications, including their side effects [27]. About a third of studies reported discussing symptoms (*n* = 8), whether patients shared their symptoms with their providers [37], or providers responded to these concerns [38] (Table 3).

**Table 3:** Topics Reported in Included Studies (*n* = 27)

First Author	Publication Year	Provider in Communication with Patient	Topics Patients Report Discussing with Provider						
			Social History	Symptoms	Explanation of Results/Disease	Management/Next Steps	Prognosis	Patient Coping Mechanisms	Other
Campesino	2012	Physician		x	x	x			Insurance
Chatman	2011	Physician; Nurse; Community Health Worker				x			Barriers to care
Check	2018	Physician			x	x			
Davey	2010	Physician				x			Navigating health system
Greene	2020	Physician; Nurse; Technician		x	x	x			
Harper	2015	Physician			x	x			
Hawley	2008	Physician			x	x			
Heiney	2017	Physician; Nurse			x	x			
Housten	2022	Physician; Nurse			x	x			
Kollie	2017	Not Specified			x	x			
Kwan	2013	Physician		x	x	x			
Livaudais	2012	Physician			x				
Manfredi	2010	Physician		x	x	x			
Samuel	2016	Physician	x	x					
Shelton	2013	Physician				x			
Sheppard	2015	Physician				x			
Sheppard	2011	Physician				x	x		
Sheppard	2010	Physician; Nurse		x		x	x	x	
Sheppard	2013	Physician				x			Patient's opinions about chemotherapy
Sheppard	2016	Physician				x			Reasons for medical tests
Song	2012	Physician; Nurse; Technician		x		x			
Sutton	2019	Physician			x				
Torres	2016	Physician			x	x		x	
White-Means	2016	Physician			x	x		x	Family reaction; Impact on life
White-Means	2017	Physician				x		x	
Wise	2008	Physician			x	x		x	
Yoon	2008	Physician		x		x			

Patient coping mechanisms (*n* = 5), family (*n* = 1) and social (*n* = 2) history, and prognosis (*n* = 2) were rarely discussed. Communication about barriers to care, such as insurance (*n* = 1), navigating the health system (*n* = 1), and patients’ opinions about chemotherapy (*n* = 1), were infrequently reported.

The communication setting, such as whether patients and providers were together in-person in an outpatient or inpatient setting, or speaking over the phone or telehealth portal, was rarely specified. Some studies included patient-report of having received their breast cancer diagnosis over a phone conversation [22, 28, 45, 46]. The mode of recorded communication, such as whether by post mail, message in online patient portal, voicemail, was rarely reported.

### Black participants’ appraisal of patient-provider communication

#### Positive appraisal of patient-provider communication

In addition to the topics discussed, the quality of communication with their providers was immensely valued by patients across the included studies. In addition to providers’ perceived expertise and others’ referrals [32], patients trust of their providers was impacted by the quality of their relationship.

One study observed that patients’ positive appraisal of their providers was based on their relational experience with them, rather than their perceived technical expertise [28]. The quality of patients’ relationship with their provider was defined by how much time they spent with them [29, 41] and how much they assured them [22]. Another study highlighted how providers gave their patients hope through sentiments such as “This time next year you are going to be saying this was a dream..you are going to get through this” [28]. Several included articles highlighted how patients felt especially supported when their providers collaborated with other experts [30, 32, 44, 45]. Patients felt comfortable discussing treatment when providers created a supportive environment and related to their patients [29]. Specifically, physicians’ acknowledgment of the role of faith in patients’ lives, as well as sharing their own spiritual beliefs, brought comfort to patients and helped them cope with their diagnosis [44, 45].

Patients perceived communication was high quality when providers explained treatment [28, 29, 32, 33, 41, 44]. Furthermore, patients often attributed these explanations as essential to preparing for side-effects [33, 44] and even preventing them from going into a depression [28]. Another study described how a patient wasn’t planning on pursuing chemotherapy until her physician explained the importance of the treatment [44]. Providers were noted for offering assistance and informational resources that helped patients understand and adhere to their treatment plans and treatment side effects [41]. Studies described how providers were sensitive to patients’ health literacy [44], such as using metaphors to facilitate patients’ understanding of the need for adjuvant therapy [32]. One study reported that patients were satisfied with the amount of information they were given to make a treatment decision [23].

Beyond providers’ communication with them, patients noted how meaningful it was when providers communicated with their relative to keep them updated on their treatment, helping patients focus on the treatment itself [41, 44].

#### Negative appraisal of patient-provider communication

Patients reported topics they wish they were told about before being diagnosed, including types of breast cancer, treatments for each type, treatment side effects, as well as broader health concerns such as egg retrieval, sexual health, early menopause [46]. A study found that patients had lower ratings of their discussion with providers on how treatment would affect their daily activities such as work [30], or non-physical side effects such as social isolation or financial toxicity [38].

In addition to topics they wished they discussed, there were several aspects of communication that were poorly perceived. Patients reported low quality of communication when providers spent little time with them, such as less than 15 minutes [29], or when providers called days later than then said they would [22]. A few studies reported that patients were traumatized to receive their breast cancer diagnosis over the phone, and would have preferred to receive the news in person [22, 28, 45, 46].

Patients also perceived low quality of communication when they didn’t understand their treatment course, treatment side effects, or possibility of recurrence [23, 27, 29, 42, 46, 47]. One study observed that patients were unfamiliar with medical terminology to describe their type of cancer or prognosis, as well as justification for treatments [23]. Another study described how patients were uninformed about essential characteristics of treatment, such as how long or debilitating it would be, or what to expect after reconstructive surgery [46]. Conversely, another study found that patients were overwhelmed by all the information they received [28].

Studies found that patients felt their providers ignored or were unaware of their attempts to report symptoms [49]. Other studies reported that patients perceived their providers ignored their views on treatment options [31]. Patients attributed their negative appraisal of patient-provider communication with discouraging them from seeking care with the provider in the future [29], interfering with their treatment initiation [22], or even prematurely withdrawing from treatment [44]. Black patients reported high decision dissatisfaction and regret [44] compared to white women [31]. Patients who reported poor patient-provider communication reported feeling less confident about their decisions about adjuvant treatment [41]. Compared to patients of other racial backgrounds, a larger proportion of Black patients reported having “not enough responsibility” for treatment decision-making [36].

Studies also found that patients perceived their providers were dishonest [44], such as having ulterior motives to recruit patients for a clinical trial [46]. Furthermore, Black patients were more likely to report medical mistrust compared to white patients [38–41]. A study found that medical mistrust was inversely related to communication ratings about radiation and chemotherapy, and significantly associated with patient report of technical quality [19]. Compared to patients of other racial backgrounds, a larger proportion of Black patients reported their provider did not know about novel treatments [49].

Patients described that when providers only showed their “professional side,” or solely discussed technical details about care, such as statistics and medications, rather than patients’ feelings [41], they didn’t feel cared for “as human beings” [28]. Furthermore, several studies described how healthcare providers made them feel hopeless, with one patient reporting that a provider said “nothing could be done” if there was a cancer recurrence [28], and there is “nothing [healthcare providers] can do for [patients]” [22]. Studies described how healthcare providers made patients feel uneasy, such as by telling patients they were “lucky to have” them [22]. Other studies reported that patients felt providers didn’t care about their “state of mind” [28], and perceived they instead cared about “getting their money” [29]. One patient described a provider who “barreled through” the appointment, making her feel like he didn’t care about her unless she was a participant in his study or “half-dead” [44].

#### Observed health-related outcomes associated with communication

In addition to patients attributing patient-provider communication to their health behaviors, some studies also collected data on the clinical outcomes associated with patient-provider communication. Fewer than half of the included studies reported health related outcomes that were associated with reported patient-provider communication (*n* = 12).

Black patients’ positive appraisal of their communication with their provider was associated with their treatment decisions. For example, patients attributed their treatment choices based on their consultation with their surgeon and oncologist [26]. The one intervention study of the included articles found that there was greater healthcare participation among Black patients when didactic information was shared [48]. Patients who reported quality communication were more knowledgeable about their treatment [41], and satisfied with their treatment decisions [41]. A study reported that patients with more positive experience with their health care providers reported self-efficacy to seek type of care to fit their needs [29].

Black patients’ positive appraisal of their communication with their provider was also associated with treatment initiation and adherence. Patients who reported quality communication with their providers were more likely to initiate treatment compared to patients who did not report quality communication [42]. Patients who reported quality communication adhered to adjuvant therapy [41], and attributed their continuation of treatment to their providers’ encouragement [44].

Conversely, a study found that Black patients stopped or delayed treatments [40] without consulting their providers due to poor communication with providers and care dissatisfaction [34], including rejecting chemotherapy [41]. Similarly, lower trust in oncologist was associated with greater time to therapy initiation [42].

Black patients’ negative appraisal of their communication with their provider was also associated with their physical well-being. A study found that Black patients' rating of “lack of clarity” in their communication with their provider was strongly negatively associated with their rating of physical well-being [27]. A study reported that a patient attributed her lymphedema to lack of communication about treatment side effects with her provider [44]. Another study reported that a patient did not receive sufficient pain relief due to lack of communication with her provider about her pain [46]. Black patients ratings of technical care were associated with perceived lack of support from providers, trust in providers, and feelings of discrimination [43]. A study found that Black patients were less likely than white patients to have received the name of a cancer expert or cancer center, even after controlling for other variables [37].

#### Highlighted directions for future research

The authors of the included studies identified next steps for future research. Many called for the need to better understand patient-provider communication, including the impact of mistrust [39] and health literacy [19]. Studies called for research of providers’ assessment of communication, to understand whether the experience was mutual [29, 43]. Several authors highlighted the need to better understand how patients integrate treatment information from multiple sources, including provider recommendations, the internet [26], and their social network [39].

Studies emphasized the need to better understand how communication can promote patient knowledge of disease, and promote patient self-efficacy [34, 40] and shared decision-making [23, 32, 36, 38, 41, 42]. Several authors underscored the need to understand the impact of communication on outcomes such as delayed treatment initiation, treatment adherence, physical, mental and emotional well-being [26, 33, 48]. Studies highlighted the need to for future research to examine communication strategies to address patient fears and concerns [20, 29], and reflect cultural sensitivity, respect and inclusion [24, 31, 32, 36].

## Discussion

Despite the high prevalence of breast cancer and the significant racial disparity in health outcomes, the healthcare services field has a limited understanding of the best way to communicate essential information to Black patients with breast cancer, who have reported high need for greater informational support. This scoping review highlights several gaps in the literature of which patient-provider communication methods are culturally appropriate for Black women with breast cancer. The limited existing evidence has demonstrated that patients perceived high quality communication when providers spent time with them, explained treatment and side-effects, and created a supportive environment such as acknowledging the role of their faith and updating the patient’s relatives. Patients perceived low quality communication when providers neglected their symptoms and failed to describe what to expect for treatment, as well as treatment’s broader impact on patient’s ability to work, or social isolation. While the included articles did not consistently measure health behaviors and outcomes in conjunction with communication measures, there is evidence that patients’ positive appraisal of communication with their provider was associated with treatment initiation, adherence and satisfaction, while patients’ negative appraisal was associated with unmanaged physical symptoms and delaying or prematurely ending treatment.

This scoping review’s results are consistent with findings from studies of patients from other racial and ethnic backgrounds. The finding of Black patients’ negative appraisal when healthcare providers avoided discussing the impact of treatment on their ability to work was reported in studies of other patient populations [50, 51]. This review found that Black patients’ perception of their healthcare providers was based on their relational experience with them, which parallels the finding that patients highlighted their physician’s personality when asked to describe their care [52]. This review demonstrated that Black patients’ perception of communication quality was associated with patient satisfaction, a finding which was also reported in a study of white and Chinese women with breast cancer [53], as well as a study of racially diverse patients with breast cancer [54]. We also found an association between Black patients’ appraisal of their communication with clinical outcomes. This association is consistent with results from a study of Hispanic patients with breast cancer [55] and a study of largely white patients with breast cancer [56], which reported an inverse relationship between patient communication ratings and symptom burden. The finding that Black patients attributed their healthcare providers’ explanation of anticipated treatment side effects as essential to managing symptoms parallels results that patients attributed their decision to take adjuvant endocrine therapy to their communication with their healthcare provider [52]. The result that Black patients’ appraisal of their communication was associated with treatment adherence is consistent with studies of other patient populations [10, 19, 52, 57].

This work highlighted findings that are unique to Black patients with breast cancer. For example, Black patients have reported more decisional dissatisfaction, regret, and medical mistrust compared to white patients. Black patients have also been less likely than white patients to have received the name of a cancer expert or cancer center. Compared to patients of other racial backgrounds, Black patients with breast cancer reported not having enough input into treatment decision-making and that their healthcare providers were not knowledgeable about novel treatments.

Several methodological limitations were noted among the included articles of this scoping review. Over half of the included studies (n = 14) failed to report the stage of patient diagnosis, and about a quarter of the studies (n = 6) included participants with diagnosis from Stage I through and inclusive of Stage IV. Failing to specify the stage of diagnosis or aggregating results of patients with varying stages of diagnosis impedes our ability to compare findings across different populations, whose needs can vary substantially. For three studies, the proportion of Black participants was less than the proportion of Blacks in the US; one study neglected to report the subset of Black participants in the sample. Oversampling of Black participants in future studies is needed to better understand the specific needs of this patient population.

The included articles also revealed limitations in data collection. Few of the included studies used validated instruments to measure patients’ perception or appraisal of the communication they received. The majority of included articles (n = 15) failed to report measured health outcomes in addition to the participant communication appraisal. Given this study’s findings that communication measures are associated with health outcomes, including patient physical well-being and treatment decisions including adherence, future studies on patient-provider communication should commit to collecting concurrent data on health outcomes. Nearly all the articles studied patient-provider communication asynchronously, subject to recall bias. There is a need, as several authors of included articles underscored, for research that directly observes communication in real-time. The included articles rarely reported the setting in which patient-provider communication took place, or whether it was live or recorded. Given evidence that patients found it highly detrimental when providers left recorded messages about their test results, the setting of communication is worth collecting to measure any variation in the prevalence and quality of messages. Such data collection is especially important, given the increasing use of electronic health portals.

Most articles reported which healthcare provider engaged in communication with patients. Collecting this data remains essential, as healthcare systems are increasingly incorporating a multi-disciplinary team that interacts with patients throughout their continuum of care. It will be critical to discern the advantages and challenges associated with different roles communicating different pieces of information, such as a physician’s assistant sharing the breast cancer diagnosis prior to patient’s first visit, or nurses sharing what to expect for future chemotherapy sessions.

This scoping review had methodological strengths, including the creation and implementation of a comprehensive, reproducible search strategy. Coders achieved reliability before independently screening the articles. This review also had limitations. Though screening of ambiguous studies was consulted with the authorship team, a single screener for full-text article selection could have introduced bias. Additionally, due to the limited studies published on this topic for this population, articles were not screened for quality in this review. As more studies focus on this population, future reviews can screen for article quality to ensure validity.

This review highlighted areas in which communication between Black patients with breast cancer and their healthcare providers can be improved. Discussions of barriers to care, family and social history, and patient coping mechanisms were rarely reported. Research is needed to examine how frequently and in what manner these topics are discussed in order to improve the quality in which they are communicated. Similarly, more research is needed to develop consistent approaches to share more medical information, especially about patients’ treatment plan, and to foster trust towards patients’ healthcare providers. Future studies are required to elucidate the information and communication needs of Black patients with breast cancer, and how they may be unique from the general population, in order for researchers to design tailored interventions that mitigate the racial disparity in breast cancer outcomes.

## Data Availability

No datasets were generated or analysed during the current study.

## Appendix A

PubMed (NLM)

Note: The African American Racial Disparities search hedge is borrowed from Chelsea Misquith, Brown University Library.

Embase (Elsevier)

Note: The African American Racial Disparities search hedge is borrowed from Chelsea Misquith, Brown University Library.

Web of Science (Clarivate Analytics)

Note: The African American Racial Disparities search hedge is borrowed from Chelsea Misquith, Brown University Library.

**Table:**

#1	TS = (african* OR “african american* “ OR black* OR “people of color “ OR “people of colour “ OR “person of color “ OR “person of colour “ OR “black people “ OR “black women “ OR “black woman “ OR POC OR “african continental ancestry group “) AND TS = ((female* OR women OR woman))
#2	TS = (mammogr* OR “3D mammogr*” OR “breast tomosynthes*” OR xeromannogr* OR “ultrasonic mammogr*” OR “mammary ultrasonograph*” OR “breast ultrasonograph*” OR “breast neoplasm*” OR “breast tumor*” OR “breast tumour*” OR “breast cancer*” OR “malignant breast*” OR “mammary cancer*” OR “cancer NEAR/3 breast*” OR “mammary carcinoma*” OR “breast carcinoma*” OR “breast NEAR/3 screen*”)
#3	TS = (“patient provider communicat*” OR “patient NEAR/3 communicat*” OR “health communication*” OR “physician patient relation*” OR communicat* OR “communication bevahior*” OR convers* OR percept* OR “truth disclos*” OR “social relation*” OR “health attitude*” OR “health knowledge” OR attitude* OR opinion* OR sentiment* OR “information disseminat*” OR “information shar*” OR “interview as topic” OR “group interview” OR interview*)
#4	TS = (“culturally competent care” OR “cultural care” OR “cross cultural care” OR “culturally sensitive care” OR “culturally congruent care” OR “culturally NEXT/3 care” OR “cultural competen*” OR “culturally sensitiv*” OR “cultural background” OR cultur* OR “culturally appropriat*” OR belief* OR custom* OR value* OR “ethnic norm*” OR “culturally relativ*” OR “culture background” OR spiritual* OR religio* OR “religious belief*” OR “religious ethic*” OR pray* OR “cultural character*” OR “cultural depriv*”)
#5	#1 AND #2 AND (#3 OR #4)

Applied Social Sciences Index and Abstracts (ProQuest).

Note: The African American Racial Disparities search hedge is borrowed from Chelsea Misquith, Brown University Library.

**Table:**

S1	TI(african* OR “african american*” OR black* OR “people of color” OR “people of colour” OR “person of color” OR “person of colour” OR “black people” OR POC OR “african continental ancestry group”) OR AB((african* OR “african american*” OR black* OR “people of color” OR “people of colour” OR “person of color” OR “person of colour” OR “black people” OR POC OR “african continental ancestry group”)) OR MAINSUBJECT.EXACT.EXPLODE("African people") OR MAINSUBJECT.EXACT.EXPLODE("African")
S2	TI(female* OR women OR woman) OR AB((female* OR women OR woman)) OR MAINSUBJECT.EXACT.EXPLODE("Females")
S3	TI(mammogr* OR “3 d mammogr*” OR “breast tomosynthes*” OR xeromannogr* OR “ultrasonic mammogr*” OR “mammary ultrasonograph*” OR “breast ultrasonograph*” OR “breast neoplasm*” OR “breast tumor*” OR “breast tumour*” OR “breast cancer*” OR “malignant breast*” OR “mammary cancer*” OR “cancer of the breast*” OR “mammary carcinoma” OR “breast carcinoma*” OR “breast screen*”) OR AB((mammogr* OR “3 d mammogr*” OR “breast tomosynthes*” OR xeromannogr* OR “ultrasonic mammogr*” OR “mammary ultrasonograph*” OR “breast ultrasonograph*” OR “breast neoplasm*” OR “breast tumor*” OR “breast tumour*” OR “breast cancer*” OR “malignant breast*” OR “mammary cancer*” OR “cancer of the breast*” OR “mammary carcinoma” OR “breast carcinoma*” OR “breast screen*”)) OR MAINSUBJECT.EXACT.EXPLODE("Mammography") OR MAINSUBJECT.EXACT.EXPLODE("Breast cancer")
S4	TI(“patient provider communication” OR “patient NEAR/3 communication” OR “health communication” OR “physician patient relation*” OR communicat* OR “communication bevahior*” OR convers* OR percept* OR “truth disclos*” OR “social relation*” OR “health attitude*” OR “health knowledge” OR attitude* OR opinion* OR sentiment* OR “information disseminat*” OR “information shar*” OR “interview as topic” OR “group interview” OR interview*) OR AB((“patient provider communicat*” OR “patient NEAR/3 communicat*” OR “health communication*” OR “physician patient relation*” OR communicat* OR “communication bevahior*” OR convers* OR percept* OR “truth disclos*” OR “social relation*” OR “health attitude*” OR “health knowledge” OR attitude* OR opinion* OR sentiment* OR “information disseminat*” OR “information shar*” OR “interview as topic” OR “group interview” OR interview*)) OR MAINSUBJECT.EXACT.EXPLODE("Doctor-Patient communication") OR MAINSUBJECT.EXACT.EXPLODE("Doctor-Patient interactions") OR MAINSUBJECT.EXACT.EXPLODE("Doctor-Patient relationships") OR MAINSUBJECT.EXACT.EXPLODE("Communication")
S5	TI(“culturally competent care” OR “cultural care” OR “cross cultural care” OR “culturally sensitive care” OR “culturally congruent care” OR “culturally NEXT/3 care” OR “cultural competency” OR “culturally sensitiv*” OR “cultural background” OR cultur* OR “culturally appropriat*” OR belief* OR custom* OR value* OR “ethnic norm*” OR “culturally relativ*” OR “culture background” OR spiritual* OR religio* OR “religious belief*” OR “religious ethic*” OR pray* OR “cultural character*” OR “cultural depriv*”) OR AB((“culturally competent care” OR “cultural care” OR “cross cultural care” OR “culturally sensitive care” OR “culturally congruent care” OR “culturally NEXT/3 care” OR “cultural competen*” OR “culturally sensitiv*” OR “cultural background” OR cultur* OR “culturally appropriat*” OR belief* OR custom* OR value* OR “ethnic norm*” OR “culturally relativ*” OR “culture background” OR spiritual* OR religio* OR “religious belief*” OR “religious ethic*” OR pray* OR “cultural character*” OR “cultural depriv*”)) OR MAINSUBJECT.EXACT.EXPLODE("Cultural attitudes") OR MAINSUBJECT.EXACT.EXPLODE("Cultural awareness") OR MAINSUBJECT.EXACT.EXPLODE("African religious customs")
S6	(1 AND 2) AND 3 AND (4 OR 5)

Cochrane CENTRAL (Wiley)

Note: The African American Racial Disparities search hedge is borrowed from Chelsea Misquith, Brown University Library

**Table:**

#1	((african*):ti,ab,kw OR (african NEXT american*):ti,ab,kw OR (black*):ti,ab,kw OR (people NEXT of NEXT color):ti,ab,kw OR (person NEXT of NEXT color) OR (black OR people) OR (POC) OR (african NEXT continental NEXT ancestry NEXT group) AND ((female OR woman OR women))):ti,ab,kw
#2	((mammogr*):ti,ab,kw OR (3D NEXT mammogr*):ti,ab,kw OR (breast NEXT tomosynthes):ti,ab,kw OR (xeromannogr*):ti,ab,kw OR (ultrasonic NEXT mammogr*):ti,ab,kw OR (mammary NEXT ultrasonograph*):ti,ab,kw OR (breast NEXT ultrasonograph):ti,ab,kw OR (breast NEXT neoplasm*):ti,ab,kw OR (breast NEXT tumor*):ti,ab,kw OR (breast NEXT tumour):ti,ab,kw OR (breast NEXT cancer*):ti,ab,kw OR (malignant NEXT breast*):ti,ab,kw OR (mammary NEXT cancer*):ti,ab,kw OR (cancer NEAR/3 breast*):ti,ab,kw OR (mammary NEXT carcinoma*):ti,ab,kw OR (breast NEXT carcinoma*):ti,ab,kw OR (breast NEXT screen*)):ti,ab,kw
#3	((patient NEXT provider NEXT communication):ti,ab,kw OR (patient NEAR/3 communication):ti,ab,kw OR (health NEXT communication):ti,ab,kw OR (physician NEXT patient NEXT relation*):ti,ab,kw OR (communicat*):ti,ab,kw OR (communication NEXT behavior*):ti,ab,kw OR (convers*):ti,ab,kw OR (percept*):ti,ab,kw OR (truth NEXT disclos*):ti,ab,kw OR (social NEXT relation*):ti,ab,kw OR (health NEXT attitude*):ti,ab,kw OR (health NEXT knowledge):ti,ab,kw OR (attitude*):ti,ab,kw OR (opinion*):ti,ab,kw OR (sentiment*):ti,ab,kw OR (information NEXT disseminat*):ti,ab,kw OR (information NEXT shar*):ti,ab,kw OR (interview NEXT/3 topic):ti,ab,kw OR (group NEXT interview):ti,ab,kw OR (interview*)):ti,ab,kw
#4	((culturally NEXT competent NEXT care):ti,ab,kw OR (cultural NEXT care):ti,ab,kw OR (cross NEXT cultural NEXT care):ti,ab,kw OR (culturally NEXT sensitive NEXT care):ti,ab,kw OR (culturally NEXT congruent NEXT care):ti,ab,kw OR (culturally NEXT/3 care):ti,ab,kw OR (cultural NEXT competency):ti,ab,kw OR (cultural NEXT background):ti,ab,kw OR (cultur*):ti,ab,kw OR (culturally NEXT appropriat*):ti,ab,kw OR (belief*):ti,ab,kw OR (custom*):ti,ab,kw OR (value*):ti,ab,kw OR (ethnic NEXT norm*):ti,ab,kw OR (culturally NEXT relativ*):ti,ab,kw OR (culture NEXT background):ti,ab,kw OR (spiritual*):ti,ab,kw OR (religio*):ti,ab,kw OR (religious NEXT belief*):ti,ab,kw OR (religious NEXT ethic*):ti,ab,kw OR (pray*):ti,ab,kw OR (cultural NEXT character*):ti,ab,kw OR (cultural depriv*)):ti,ab,kw
#5	#1 AND #2 AND (#3 OR #4)

MedRxiv (https://www.medrxiv.org/).

TRIP Pro medical database (tripdatabase.com).

## Appendix B

Initial searching yielded the following results: PubMed (NLM) *from inception to 6/13/2022 (n* = 2,077), Embase (Elsevier) *from inception to 6/13/2022 (n* = 3,761), Web of Science (Clarivate Analytics) *from inception to 6/13/2022* (n = 2,188), Applied Social Sciences Index and Abstracts (ProQuest) *from inception to 6/13/2022* (n = 276), Cochrane Central (Wiley) *from inception to 6/13/2022* (n = 516).
